# Mr. Henry Dobbin

**Published:** 1893-10-28

**Authors:** 


### Hospital Worthies.

### Mr. Henry Dobbin.

On October 1st the secretarial duties of the Brompton Hospital for Consumption were taken over by Mr. W. H. Theobald, for many years assistant secretary, on the retirement of Mr. Henry Dobbin on a pension.

We called attention to the services of Mr. Dobbin in our issue of August, 19th, 1893, but we desire now to give a short statement of what he has done for the Brompton Hospital during the thirty-five and a-half years of his connection with it.

The Brompton Hospital unlike, most other metropolitan hospitals, has an hon. secretary. The first hon. secretary was Mr. Philip, afterwards Sir Philip, Rose, who founded the hospital and rendered such important services to the institution that a "Rose Fund" was raised to commemorate them. The Rose Fund now amounts to upwards of £2,100; the income of this fund, about £200 per annum, is devoted to the relief of patients by gifts of clothes and small pecuniary assistance on their leaving the hospital.

The Committee of the Brompton Hospital, of which Mr. Beckwith is the able chairman, has always been commendably active and helpful. Mr. Dobbin has had the valued and loyal co-operation for many years of Mr. Theobald, who now succeeds him, and the inherent value and merit of the charity itself has from the outset secured an increasing volume of sympathy and support. We mention these facts at the outset, in order to do justice to all who are entitled to claim credit in connection with this splendid institution which now ministers to the needs of those who suffer from consumption and diseases of the chest.

When due credit has, however, been extended to everybody, the fact remains that to Mr. Dobbin must be given the largest meed of praise for results which it is here our pleasant duty to chronicle. Mr. Dobbin was appointed secretary in succession to Mr. Cross, and took up his duties on March 26th, 1858. At that time the funds of the hospital were so low that the committee was, with great reluctance, compelled to close an entire wing of the building containing 80 beds. In consequence of this step the in-patients in the hospital were reduced from 169 on December 31st, 1856, to 114 at the same date in the following year. The committee had also to face a considerable falling off in various sources of income, and they were on December 31st, 1857, practically without any invested property, as the dividends amounted to less than £4 a-year, though the ground rents from the hospital estate yielded an income of £226. The annual subscriptions amounted to £3,358, the donations to £3,472, the legacies to £862, the charity sermons to £304, and the total annual income from all sources to £8,334, against an expenditure under all heads of £8,410. The hospital had at that time about 200 available beds.

On December 31st, 1892, the income from dividends was £3,970, in addition to £1,392 from ground and other rents, or together £5,362; the annual subscriptions amounted to upwards of £8,000 per annum; donations to upwards of £4,626, legacies to £12,973, church collections, &c., to £2,190. In addition to these splendid resources, incidental receipts, which include £3,476 from private nursing, and £27 from alms boxes, amounted to £3,591, the total receipts from all sources being £36,793, against a total expenditure of £30,593. The hospital at the present time contains 347 beds, and its list of governors and subscribers constitutes about the best and strongest subscription list of any medical charity in the world.

These facts constitute a monument to fruitful labours which must confer honour on the name of Henry Dobbin for all time. It has been well said that finance is the key to success in worldly matters. Certainly, Mr. Dobbin and those who have worked with him have good cause to claim that they have recognised the force of this statement, and have so achieved a marvellous success.

The bare recital of facts like the foregoing will not, however, do anything like justice to the work which has been accomplished during the past 35 years at Brompton. The staff of assistant physicians in charge of the out-patients has been doubled; the number of out-patients under treatment during 1857 having been 4,500, against 13,357 in 1892. The average number of beds occupied has increased from 114 in 1857 to 270½ during 1892. Thus the committee are justified in claiming that not only does their hospital afford relief to multitudes of indigent persons suffering from so terrible and mysterious disease as that of consumption, but that they have the reflex benefit afforded to themselves and others by the improved professional knowledge of a malady which is no respecter of persons or circumstances. There are, alas! far too few hospital beds available for cases of consumption in England to-day, but the misery and suffering caused by the paucity of accommodation would have been immensely increased had it not been for the life-work of Mr. Dobbin and his colleagues, who have succeeded in more than doubling the accommodation for this class of sufferers which they found available when they first took charge of the Brompton Hospital.

The original Hospital for Consumption was a handsome building replete with much that was comfortable, and containing many features which made it remarkably efficient, having regard to the date of its erection. The new extension building, the foundation stone of which was laid by their

Royal Highnesses the Prince and Princess of Wales, contains a spacious out-patient department, and is fitted with every modern appliance which can conduce to the comfort and well-being of the patients. The committee were wise enough to remove the kitchen to the top of the building, and so to set an example which it would be well for the patients if every other hospital committee were to follow. The cost of the site and of the new buildings was so large as to absorb the greater portion of the funded capital, including the late Miss Read's bequest. The financial condition, therefore, in 1881-2, when the new building was opened, was highly critical. Not only had the income arising from the invested property been lost, but the committee had to face additional expenses for the yearly maintenance of the institution, which amounted to £10,000 per annum. We mention these facts to show that Mr. Dobbin commenced his secretarial duties with a financial deficiency which was so serious as to necessitate the closing of 80 beds; that in process of time he accumulated funds and raised an income so large as to induce the committee to expend the whole of the invested funds in new buildings, without making any appeal whatever for contributions to a building fund. During the last eleven years, under the exceptionally able financial administration which has marked the career of the Brompton Hospital, the invested property has crept up to a sum which brings in an income, as we have already shown, of upwards of £5,000 a year; and the hospital now expends upwards of £24,000 a year on maintenance, of which £19,000 is annually raised by voluntary contributions. This is a truly splendid record in the history of British charities, which entitles all concerned to receive the gratitude and recognition of all classes of their countrymen.

So much for the work. Let us now consider the man himself. Kindly, courteous, a master of the details of his work, and immensely interested in it, Mr. Dobbin represented in his own person the best type of hospital secretary. Nothing seemed a trouble, especially if it would tend in any way to relieve a sufferer, or to help anyone who was interested in the work. It would be impossible to give an account of the many improvements and additions to the comfort of the patients which owe their origin to Mr. Dobbin. We will mention only one. Recognizing that consumptive patients as a class are not too ill to be amused, Mr. Dobbin set himself to organise a plan which should tend to aid recovery by providing a diversion for the patients, and so inculcate cheerfulness. Twenty-six years ago he organised the weekly entertainments for patients, which have been continued every winter since, from November to April. The idea was a good one. the splendid hall in the new building has given them a character all their own, and we could wish that the example here set might be widely followed, especially at convalescent institutions and chronic hospitals. Had a man of Mr. Dobbin's character and powers devoted his life to business he would probably have realized a fortune, and, at any rate, have enjoyed a substantial income. There are an increasing number of ill-informed critics who never weary in their abuse of hospital officials on the ground of the excessive remuneration which they receive. If such persons would only pause and ascertain the facts, astonishment would seal their lips for ever. Mr. Dobbin is a case in point. He commenced his career as secretary at Brompton Hospital with a salary of £300 per annum, served the institution faithfully for thirty-five and a-half years, during which time the institution thrived exceedingly, as it has been our pleasant duty to prove, and on relinquishing office his income did not exceed £500 a-year.

Here is precise evidence that those who devote their lives to the administration of hospitals do not take up the work for pecuniary reasons, at any rate. They work hard, they not infrequently die in harness, or when a grateful meeting of governors gives them a moderate pension they may have to submit to the unjust and cruel taunts of irresponsible busy-bodies, who endeavour to injure the charity which may owe its well-being in great measure to the devotion of the particular officer concerned, by denouncing all pensions whatsoever. *Mirabile dictu!* Englishmen must nowadays be made of poor stuff indeed, if the next meeting of governors so assaulted does not rise in indignation, and consign any such critics to the nearest horsepond.

If ever men earned and deserved pensions, those men are Mr. W. J. Nixon, of the London Hospital, and Mr. Henry Dobbin. They are "Hospital Worthies," who have done splendid service in the cause of suffering humanity, and we make no doubt that in due time they will reap a reward which none can tarnish nor take away.

